# Novel MDR-reversing inhibitors of MRP subtypes related to antiretroviral drug resistance and CD4 cell counts

**DOI:** 10.1186/1471-2334-14-S2-P77

**Published:** 2014-05-23

**Authors:** Andreas Hilgeroth, Paul Naujoks, Sebastian Neuber, Marc Hemmer, Hermann Lage, Joséf Molnar

**Affiliations:** 1 Institute of Pharmacy, Martin Luther University Halle-Wittenberg, Halle, Germany

## Introduction

Transmembrane efflux pumps play an increasing role in the success of ART. The occurrence of increasing mRNA levels of the efflux pump proteins P-gp and MRP subtypes has been associated with ART therapeutics like PIs and NNRTIs. Moreover, a relation has been suggested of MRP subtypes and CD4 cell counts. So there is a challenge to find selective MRP inhibitors which may reduce MRP-induced drug resistances as well as discussed effects on CD4 cells. We developed novel nonpeptidic inhibitors of important MRP subtypes related to both MRP-mediated resistances as well as reduced cell counts.

## Materials and methods

The molecular structures of the various efflux pumps guided the development of both symmetric and nonsymmetric inhibitors dedicated to selectively inhibit the relevant MRP-subtypes. Inhibiting properties were evaluated in exclusively P-gp as well as MRP-overexpressing cell lines in comparison to nonexpressing parental cell lines with fluorescent substrates using flow cytometry.

## Results

Our inhibitors with allover symmetric substitution patterns are excellent P-gp inhibitors, while a reduction of those symmetric elements concerning their number and exact positioning significantly lowers the P-gp inhibition whereas the MRP1 inhibition is found mainly increased. So selective inhibitors are demonstrated to influence the efflux pump type-related antiretroviral drug resistance.

## Conclusions

The success of ART may be mainly increased by selective MRP inhibitors which reduce the MRP-related effects on drug resistance as well as on discussed cell counts. Structure-guided studies of the efflux pump inhibition helped to design novel selective inhibitors with structure-reasoned effects of the nonsymmetric substitution patterns. So far only nonselective inhibitors have been used in ART with undesired effects on transporter-related pharmacokinetics. Selective inhibitors will mean a progress in ART especially with less limiting side effects.

